# Effects of preconception counseling on maternal health care of migrant women in China: a community-based, cross-sectional survey

**DOI:** 10.1186/s12884-015-0485-4

**Published:** 2015-03-06

**Authors:** Xiaofang You, Hui Tan, Shiyun Hu, Jianmei Wu, Hong Jiang, Aiping Peng, Yue Dai, Ling Wang, Sufang Guo, Xu Qian

**Affiliations:** Department of Maternal and Child Health, School of Public Health, Key Laboratory of Public Health Safety, Ministry of Education, Fudan University, Mailbox 138, No. 138 Yi Xue Yuan Road, Shanghai, 200032 People’s Republic of China; Pinghu Institute of Maternal and Child Health, Zhejiang Province, China; United Nations Children’s Fund China Country Office, Beijing, China

**Keywords:** Maternal health care, Preconception counseling, Quality prenatal care, Folic acid supplements, Migrant

## Abstract

**Background:**

Migrants have long been a disadvantaged group in China’s health care system, especially in terms of maternal health care. Many studies have explored the factors associated with a lack of maternal health care and found many determinants, including social, economic, behavioral, and environmental factors. However, studies focusing on factors associated with maternal health care have rarely examined preconception counseling (PCC). This study explored factors related to PCC uptake among migrant women, and investigated the association between PCC and maternal health care in migrant women.

**Methods:**

A community-based cross-sectional study was conducted from July to December 2011, in Nanhai, Guangdong Province, and Pinghu, Zhejiang Province, China. A total of 1,012 migrant women who had their most recent pregnancy within 1 year of the survey answered a standardized interviewer-administered questionnaire about maternal health care. Descriptive statistics and multivariable logistic regression were used to analyze the data.

**Results:**

Only 208 (20.6%, 95% confidence interval [CI]: 18.1–23.1%) of 1,012 migrant women had received PCC. Younger age, having more than one child, lack of knowledge of maternal health care and inter-province migration were predictors of a lack of PCC. PCC was associated with higher consumption of folic acid supplements during the preconception period (adjusted odds ratio [AOR] = 2.65, 95% CI: 1.66–4.23). Among migrants who were resident in Nanhai or Pinghu for less than 5 years, PCC was related to better quality prenatal care (AOR = 3.07, 95% CI: 1.79–5.24).

**Conclusions:**

The prevalence of PCC among migrant women was low (20.6%, 95% CI: 18.1–23.1%). Positive associations were found between the receipt of PCC and preconception folic acid supplements and quality prenatal care. Future studies focusing on maternal health care should pay attention to PCC and explore the effects of PCC on maternal health care through intervention studies. Continued efforts to increase PCC in migrants should target specific age groups (20–24 years), families with more than one child, and women who have migrated between provinces, as well as provide in-depth knowledge of maternal health care.

## Background

China has entered a period of active population migration due to rapid industrialization and urbanization [1]. According to China’s State Statistics Bureau, the number of internal migrants living and working in urban areas without formal urban household registration status increased dramatically within the past few decades, reaching 220 million in 2010 [2]. Meanwhile, more women and children have started to migrate in recent years, because the migration style has changed to a “family style” in which families relocate together [3]. Zhejiang and Guangdong are the two provinces with the highest population of migrants in China, and the number of migrants in these provinces has rapidly increased in recent years. According to China’s Sixth National Population Census in 2010, there were almost 31.3 million migrants in Guangdong Province, accounting for 30.0% of its residents, and more than 11.8 million migrants in Zhejiang Province, accounting for 21.7% of its residents [2]. Situated in the center of Guangdong Province, Nanhai District borders Guangzhou, the province’s capital. The total population of Nanhai was 2.59 million, including 1.11 million migrants (42.9%), in 2011 [2]. The Pinghu Municipality of Zhejiang Province is located in its eastern section and borders Shanghai. According to the statistics reported in the census, the total population of Pinghu was 0.788 million in 2011, including 0.303 million migrants (38.5%) [2]. At the same time, both in Nanhai and in Pinghu, migrant women of childbearing age (15–49 years) accounted for more than half the number of childbearing women among the residents of those provinces.

Migrants have long been a disadvantaged group in China’s health care system, especially in terms of maternal health care, despite their large numbers [4]. The migrant population fares worse than the urban population on almost every indicator of maternal health [5]. A significant body of research has explored the factors associated with the lack of maternal health care in migrants and found many related determinants, including social, economic, behavioral, and environmental factors [4,6-8]. Lower economic status and educational level are often viewed as significant barriers to equal rights to maternal health care [8,9]. Lack of health insurance is directly associated with low use of maternal health care services [10]. Duration of residence is also related to the use of maternal health care services, as reported by Kusuma et al. [6]. Other factors, such as knowledge of maternal health care, affect the use of maternal health care services in migrants [11]. However, only a limited number of studies focusing on the factors associated with maternal health care have extended their research to include preconception counseling (PCC).

The purpose of PCC is to identify and modify the risks related to maternal health and pregnancy outcomes prior to pregnancy [12]. In China, the Ministry of Health launched The Job Specification of Preconception Care (Trial) in 2007, which elucidated the content of preconception care and introduced how to implement this service [13]. In 2011, the latest official guideline for preconception health care was developed and released by the Chinese Medical Association [14]. In the same year, the Ministry of Health issued Management Practices and Norms of Maternal Health Care, which included preconception care [15]. Couples who intended to conceive were recommended to attend hospitals for preconception counseling and risk assessment of pregnancy. However, the preconception care services were not compulsory. Detailed assessment of the needs and current situation of preconception care in China has seldom been conducted [16].

Previous studies found that PCC had positive effects on maternal lifestyle, diet, management of chronic medical conditions, and up-to-date vaccinations [17-20]. However, limited research has explored the association between PCC and maternal health care. Moreover, the factors associated with PCC have not received an adequate amount of attention from the research community. The purpose of this study was to explore factors related to PCC, and investigate the association between PCC and maternal health care through an appropriate research design, a high-quality questionnaire, and fieldwork in Nanhai and Pinghu.

## Methods

### Study design

This study is part of the Maternal and Child Health Care Service for Urban Migrants Project (2011–2015), a joint effort of UNICEF and the Chinese National Health and Family Planning Commission (formerly, the Ministry of Health), which focuses on maternal and child health care services for migrants. The project was conducted by the Department of Maternal and Child Health of the School of Public Health of Fudan University, from July to December 2011. The design was a community-based, cross-sectional survey that used an interviewer-administered questionnaire to assess the mothers of children who were less than 1 year of age.

### Study participants

The data were from the Maternal and Child Health Care Service for Urban Migrants Project (2011–2015). The project was intended to assess health status, service use of and health care management for migrant children less than 3 years of age and maternal health care of migrant women with children less than 1 year old. This study was focused on maternal health care of migrant women with children under 1 year old. “Migrant women” were defined as women with children younger than 1 year old (born after December 1, 2010), with neither of the couple having a “Hukou” (China’s household registration system) in Nanhai or Pinghu, and who had been residing in Nanhai or Pinghu for at least 6 months.

**Figure 1:**
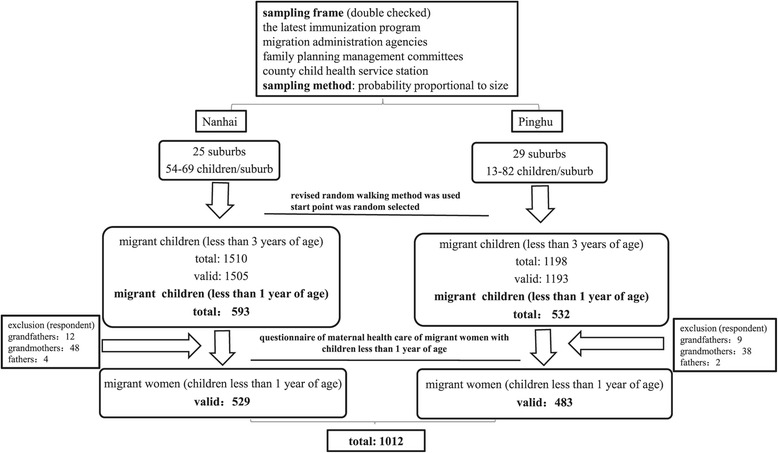
**Migrant women included in the study.**

The whole project used a multi-stage sampling method based on a World Health Organization (WHO)-advocated cluster sampling design to select eligible participants [21]. All 272 suburbs within 15 communities (still named town or township) in Nanhai and 193 suburbs within 10 communities in Pinghu served as clusters. Each Resident Administrative Committee, which was the smallest governmental administration agency, compiled the list of eligible children in their areas. Information about migrant children was mainly obtained from the latest immunization program and was then double-checked with the records from migration administration agencies, family planning management committees and the county child health service station. Probability proportional to size was used to select suburbs, and a revised random walking method with random start points was used to select children in each suburb. Finally, a total of 1,012 migrant women were included in this study (Figure 1).

To analyze potential factors (14 variables of 4 types) associated with PCC, a sample size of 704 was required based on a previous study reporting that 12.0% of the population received PCC [22], with a limit of ±5% for the 95% confidence interval (CI; α = 0.05, d = 0.2 p). Finally, we used 1,012 migrant women to analyze factors associated with PCC and to investigate the association between PCC and maternal health care.

### Data collection

The survey was divided into two phases: the community mobilization and questionnaire interview phases. Community mobilization was implemented in selected resident sampling units by community coordinators, who included individuals responsible for migrant management, cadres from the women’s federation, village doctors, and community leaders. The recruitment of the community members was conducted two to three days prior to the administration of the survey. Information regarding the eligible participants was recorded. The mothers of the migrant children under 1 year of age were encouraged to meet with the interviewers in community health centers. The eligible participants who did not attend their community health center appointments were interviewed during home visits. Interviewers (physicians from local maternal–child health centers and community health centers) and quality controllers (lecturers and senior post-graduate students from the School of Public Health of Fudan University) were trained 1 week in advance of these meetings with participants. The design of the questionnaire was based on the Maternal, Newborn, and Child Health Household Survey Guidelines (WHO, [21]). The informed consent form was adapted from a template from the Fudan University School of Public Health. The questionnaire contained items on the demographic characteristics of the children and their mothers, fathers, and families (such as the date of arrival in Nanhai or Pinghu and the Hukou or household location), in addition to knowledge of maternal health care, PCC, preconception folic acid supplements, and prenatal care use during pregnancy.

### Measures

#### Main outcome variables

The use of folic acid supplements during preconception and quality prenatal care were measured to assess the use of maternal health care services and were regarded as outcomes. PCC was used as an outcome variable when exploring associated factors, while it was a study variable when investigating the association between PCC and maternal health care.

Preconception use of folic acid supplements was measured by the question, “Did you take a folic acid supplement during the 3 months before conception?” Responses were coded as “yes” or “no”. Quality prenatal care was defined as that which included three aspects: at least five prenatal check-ups, each visit at an appropriate time, and with the recommended testing items of physical examination based on Management Practices and Norms of Maternal Health Care developed by the Ministry of Health [15,23]. Each visit at an appropriate time means one visit having occurred during the first trimester, two in the second, and two in the third (1-2-2 frequency). Recommended testing items of physical examination include weight, blood pressure, routine blood work, and urinalysis, which are documented after 36 weeks of pregnancy during at least one visit. PCC was measured by a question, “Before your latest baby was conceived, did you consult with a doctor, nurse, or other health care provider about pregnancy?” Responses were coded as “yes” or “no”.

### Study variables

The study variables included 14 variables of 4 types: demographic variables, and variables related to characteristics of the family, migrant and other. Variables related to demographic characteristics included the women’s education (elementary and below, junior high, and senior high and above), the women’s household registration (non-agriculture and agriculture), the women’s age when giving birth (<20 years, 20–24 years, and ≥25 years), the women’s occupation (whether in paid employment at the time of the survey), the fathers’ occupation (self-employed businessmen, company staff/service staff and professional/technical including officials, doctors and teachers), and the children’s sex and age.

Variables measuring the families’ characteristics included family structure (nuclear family, extended, and other), sibling/s, and family income. Family income was based on annual family income of local permanent residents and categorized by P_25_ (RMB 30,000 Yuan for Nanhai and RMB 50,000 Yuan for Pinghu) and P_75_ (80,000 Yuan for Nanhai and RMB 100,000 Yuan for Pinghu) (percentile) (≤P_25_ as lower, P_25_–P_75_ as moderate, and > P_75_ as higher). Variables related to migrant characteristics included duration of residence and migrant flow area (within provinces and between provinces).

Other variables included women’s health insurance (present or absent) and knowledge of maternal health care. There were two questions to measure knowledge of maternal health care: appropriate times for prenatal care, and symptoms of anemia during pregnancy. The answer to appropriate times was scored as “50” for correct and “0” for wrong. The question measuring symptoms of anemia comprised six parts. The answer to each part was scored as “8.33 (50/6)” for correct and “0” for wrong [24]. Total knowledge score was equal to the two scores added together, and ranging from 0 to 100 (Table 1).

**Table 1:** **Definition of variables used in the analysis**

**Variable**	**Values**
**Dependent variables**	
Preconception use of folic acid supplements	1 = Yes 0 = No
Quality prenatal care	1 = Yes 0 = No
PCC	1 = Yes 0 = No
**Study variables**	
*Variables related to demographic characteristic*	
Women’s education	1 = Senior high and above 2 = Junior high 3 = Elementary and below
Childbearing age (years)	1 = “≥25” 2 = “20–24” 3 = “<20”
Household registration	1 = Agriculture 0 = Non-agriculture
Women’s occupation	1 = Employed 0 = Not employed
Father’s occupation	1 = Professional/technical including officials, doctors and teachers 2 = Company staff/service staff 3 = Self-employed businessmen
Children’s sex	1 = Female 0 = Male
Children’s age (months)	1 = “6-11” 0 = “<6”
*Variables measuring families’ characteristic*	
Families’ structure	1 = Nuclear family 2 = Extended 3 = Other
Families’ income	1 = Higher 2 = Moderate 3 = Lower
Sibling/s	1 = Yes 0 = No
*Variables related to migrant characteristic*	
Migrant flow area	1 = between- provinces 0 = Within provinces
Duration of residence (years)	1 = “≥5” 0 = “<5”
*Other variables*	
Knowledge of maternal health care	Continuous variable: 0-100
Health insurance	1 = Yes 0 = No

### Quality control

To ensure the study’s quality, a thorough planning process was conducted prior to its implementation and fieldwork, beginning from the design stage. We also took periodic measures of our progress throughout the study to ensure that we were meeting the goals we set during the planning process.

During the design stage, the survey protocol was discussed and revised during several rounds of meetings with content experts, multidisciplinary professors, scholars, managers, and health service providers. Before the start of the study, we pilot tested and refined the questionnaire’s language, logic, and sequence of questions.

Prior to the implementation of the study, staff and personnel were selected based on their senses of responsibility, medical experience/background, and availability to work within the research schedule. After their selection, the interviewers were recruited from the community health centers, town hospitals, and district maternal health centers. The quality control personnel were recruited from Fudan University. Training was provided prior to the implementation of the survey.

During the field data collection, we succeeded in increasing the response rates by conducting interviews during weekends, when people were available to participate, and by selecting survey sites near the participants’ locations to reduce their travel time as much as possible. An audit of the questionnaire was conducted to increase its validity, with verification procedures that included self-checks by interviewers, audits by quality control staff, on-site inspections, and phone reviews. Quality control personnel received daily reports of the progress and quality of the questionnaires while providing timely feedback, instructions, and follow-up training to group leaders and interviewers.

### Statistical analysis

First, we calculated the prevalence and 95% CIs of women who reported receiving PCC. Second, we explored factors associated with receipt of PCC by computing the unadjusted and adjusted prevalence odds ratios and 95% CIs through logistic regression analysis. In conducting a multivariable logistic regression model, we initially retained all variables that were significant at *P* < 0.25 in the univariate analysis, or were identified as predicators of lacking PCC in the literature [25,26]. An examination of the correlations of all the variables in the final model revealed that multi-collinearity was not present.

Finally, we also investigated the association between receiving PCC and preconception use of folic acid supplements and quality prenatal care, after controlling for several confounders (including the mothers’ education, household registration, the fathers’ occupation, family income, duration of residence, migrant flow area, and knowledge of maternal health care). We also examined whether the associations between receiving PCC and each of the outcome variables pertaining to maternal health care were modified by duration of residence or knowledge of maternal health care. Stratum-specific estimates were also reported where significant effect modification was detected (i.e., duration of residence for quality prenatal care).

All statistical analyses were performed using the Statistical Package for the Social Sciences (SPSS) 16.0 (SPSS Inc., Chicago, IL, USA). A two-tailed *P* value <0.05 was considered statistically significant.

### Ethics

The protocol and all of the study instruments were reviewed and approved by the Ethics Committee (IRB) of the School of Public Health of Fudan University prior to the fieldwork. All of the participants signed the consent forms and were interviewed anonymously. They were assured that the information collected was for research purposes only and would remain confidential. The staff members signed confidentiality agreements after receiving an explanation of the purpose of the study.

## Results

### Participant characteristics and prevalence of receipt of PCC

Of the 1,012 migrant women surveyed in the study, only 208 (20.6%, 95% CI: 18.1–23.1%) had received PCC. The majority of women were older than 25 years old when they gave birth (50.3%), had a junior high school degree (64.1%), no job (74.6%), and had agricultural household registration (92.9%). Most women lacked health insurance (64.9%). More than half of the families had a nuclear structure (56.2%) with one child (65.5%). Nearly half of the families had a lower income (48.3%). More than half of the children were male (55.9%) and aged 6 to 11 months (53.3%). The majority of women lived in Nanhai or Pinghu for less than 5 years (66.8%), and tended to migrate between provinces (84.4%; Table 2).

**Table 2:** **Percentages and estimates of odds ratios for receipt of PCC in univariate and multivariable models**

			**Total sample**		**Receipt of PCC**					
			**N**	**%**	**n**	**%**	**COR**	**95% CI**	**AOR**	**95% CI**
**Maternal characteristics**	Education	Senior high and above	159	15.7	42	26.4	1.00	-	1.00	-
		Junior High	648	64.1	128	19.8	0.68	0.46-1.02	1.18	0.71-1.96
		Elementary and below	204	20.2	41	20.1	0.67	0.41-1.10	0.98	0.50-1.92
	***Childbearing age (years)***	**≥25**	**502**	**50.3**	**119**	**23.7**	**1.00**	**-**	**1.00**	**-**
		**20–24**	**403**	**40.4**	**73**	**18.1**	**0.71**	**0.51-0.99** ^*^	**0.65**	**0.42-0.99** ^*^
		**<20**	**93**	**9.3**	**16**	**17.2**	**0.68**	**0.38-1.21**	**0.65**	**0.31-1.36**
	Household registration	Non-agriculture	71	7.1	23	32.4	1.00	-	1.00	-
		Agriculture	932	92.9	183	19.6	0.50	0.30-0.85^*^	0.60	0.32-1.15
	Occupation	Employed	257	25.4	63	24.5	1.00	-	1.00	-
		Not employed	755	74.6	148	19.6	0.77	0.55-1.08	0.83	0.54-1.26
	Health insurance	Yes	289	35.1	63	21.8	1.00	-	1.00	-
		No	534	64.9	112	20.9	0.93	0.66-1.30	1.04	0.70-1.54
**Paternal characteristics**	Occupation	Professional/technical including officials, doctors and teachers	30	3	11	36.7	1.00	-	1.00	-
		Company staff/service staff	729	72.1	150	20.6	0.44	0.21-0.95^*^	0.53	0.22-1.28
		Self-employed businessmen	252	24.9	49	19.4	0.41	0.18-0.92^*^	0.40	0.16-1.00
**Families’ characteristics**	Structure	Nuclear family	569	56.2	128	22.5	1.00	-	1.00	-
		Extended	329	32.5	64	19.5	0.85	0.61-1.19	0.88	0.58-1.34
		Other	114	11.3	19	16.7	0.71	0.42-1.20	0.73	0.38-1.39
	Income	Higher	94	9.5	23	24.5	1.00	-	1.00	-
		Moderate	418	42.2	96	23.0	0.91	0.54-1.54	0.65	0.36-1.17
		Lower	478	48.3	90	18.8	0.70	0.42-1.18	0.58	0.31-1.07
	***Sibling/s***	**No**	**662**	**65.5**	**144**	**21.8**	**1.00**	**-**	**1.00**	**-**
		**Yes**	**348**	**34.5**	**67**	**19.3**	**0.86**	**0.62-1.19**	**0.58**	**0.39-0.89** ^*^
**Children’s characteristics**	Sex	Male	565	55.9	121	21.4	1.00	-	1.00	-
		Female	446	44.1	90	20.2	0.90	0.66-1.22	0.82	0.57-1.17
	Age (months)	<6	473	46.7	103	21.8	1.00	-	1.00	-
		6–11	539	53.3	108	20.0	0.91	0.67-1.23	0.83	0.58-1.19
***Knowledge of maternal health care***			**910**	**-**	**-**	**-**	**1.29**	**1.16-1.44** ^*^	**1.30**	**1.16-1.47** ^*^
**Migrant characteristics**	***Migrant flow area***	**Within provinces**	**158**	**15.6**	**49**	**31.0**	**1.00**	**-**	**1.00**	**-**
		**Between provinces**	**854**	**84.4**	**162**	**19.0**	**0.51**	**0.35-0.75** ^*^	**0.59**	**0.37-0.93** ^*^
	Duration of residence (years)	<5	664	66.8	138	20.8	1.00	-	1.00	-
		≥5	330	33.2	68	20.6	0.99	0.71-1.37	0.94	0.63-1.40

COR = crude odds ratio, AOR = adjusted odds ratio, PCC = preconception counseling.

*Statistically significant at *P* < 0.05.

### Characteristics associated with receipt of PCC

Table 2 presents the characteristics associated with receipt of PCC among the migrant women with children less than 1 year of age. Childbearing age, the number of children in a family, knowledge of maternal health care, and migrant flow area were all associated with PCC. Younger women (20–24 years old) were less likely to receive PCC compared with women older than 25 years of age (adjusted odds ratio [AOR] = 0.65, 95% CI: 0.42–0.99). Compared with the families with only one child, those with more than one child were less likely to receive PCC (AOR = 0.58, 95% CI: 0.39–0.89). Knowledge of maternal health care was associated with receiving PCC. Women who had a better knowledge of maternal health care were more likely to receive PCC (AOR = 1.30, 95% CI: 1.16–1.47). Women who had migrated between provinces had lower odds of receiving PCC than women who had migrated within the same province (AOR = 0.59, 95% CI = 0.37–0.93). Women’s household registration and fathers’ occupation were associated with receiving PCC in univariate models, but the associations were not statistically significant in the multivariable model (Table 2).

### Effects of PCC on maternal health care

In multivariable modeling, receiving PCC was significantly associated with the preconception use of folic acid supplements (AOR = 2.65, 95% CI = 1.66–4.23) and quality prenatal care (AOR = 1.58, 95% CI = 1.25–2.90). The association between PCC and quality prenatal care was modified by duration of residence. Among migrant women who were resident in Nanhai or Pinghu for less than 5 years, the association between PCC and quality prenatal care was strengthened (AOR = 3.07, 95% CI = 1.79–5.24). However, among women who were resident in Nanhai or Pinghu for more than 5 years, receiving PCC was not significantly associated with quality prenatal care (AOR = 0.91, 95% CI = 0.44–1.90; Tables 3 and 4).

**Table 3:** **Association between PCC and preconception folic acid supplements and quality prenatal care**

	**Preconception folic acid supplement**			**Quality prenatal care**		
	**N (%)**	**AOR** ^**a**^	**95% CI**	**N (%)**	**AOR** ^**b**^	**95% CI**
Receipt of PCC						
No(n = 801)	80(10.0)	1.00	-	226(30.8)	1.00	-
Yes(n = 208)	56(26.9)	2.65	1.66–4.23^*^	84(43.3)	1.58	1.25–2.90^*^

AOR = adjusted odds ratio, PCC = preconception counseling.

^a^Adjusted for mother’s education, household registration, fathers’ occupation, family income, duration of residence, migrant flow area, and knowledge of maternal health care.

^b^Adjusted for mother’s education, household registration, fathers’ occupation, family income, duration of residence, migrant flow area, knowledge of maternal health care, hospital of initiation of prenatal care, and reimbursement of prenatal care.

*Statistically significant at *P* < 0.05.

**Table 4:** **Association between PCC and quality prenatal care by duration of residence**

	**Quality prenatal care**		
	**N (%)**	**AOR** ^**a**^	**95% CI**
Duration of residence (<5 years), Receipt of PCC			
No(n = 526)	116(22.1)	1.00	-
Yes(n = 136)	55(40.4)	3.07	1.79–5.24^*^
Duration of residence (≥5 years), Receipt of PCC			
No(n = 262)	104(39.7)	1.00	-
Yes(n = 67)	28(41.8)	0.91	0.44–1.90

AOR = adjusted odds ratio, PCC = preconception counseling.

^a^Adjusted for mother’s education, household registration, fathers’ occupation, family income, area of migration flow, knowledge of maternal health care, hospital of initiation of prenatal care, and reimbursement of prenatal care.

*Statistically significant at *P* < 0.05.

## Discussion

This study found that overall only 20.6% (95% CI: 18.1–23.1%) of migrant women talked with a doctor, nurse, or other health care worker before becoming pregnant. Younger women (20–24 years), families with more than one child, women who lacked knowledge of maternal health care, and women who had migrated between provinces had lower odds of receiving PCC. After adjusting for multiple socio-demographic, reproductive, and migrant confounders, PCC was positively associated with the use of preconception folic acid supplements. Among migrant women who were resident in Nanhai or Pinghu for less than 5 years, PCC was significantly related to quality prenatal care.

In China, the existing preconception health care network is under the administration of the National Committee of the Family that was originally established for the implementation of the one-child policy. Contraception assistance used to be its first priority. Preconception health care was introduced to the system after the year 2000, and the national general guideline for providing preconception health care was issued in 2007 and then renewed in 2011 [13,14]. Although the government provided preconception care free of charge, the service was not compulsory. There has not been any assessment of how many people actually used this service nationally. According to a previous study conducted in 2012 in Fengxian District, Shanghai, few women reported receiving PCC. In that study, only 12.0% of women received PCC [22]. The prevalence (20.6%) found in our study was slightly higher than that of Shanghai. In our study, women who answered “Yes” to the question “Before your latest baby was conceived, did you consult with a doctor, nurse, or other health care provider about pregnancy?” were requested to reply with the exact time when the consultation happened. This requirement may have led to an underestimate of the real prevalence of PCC.

A particularly low rate of PCC was reported among the younger women (20–24 years) in our study, and was associated with families with more than one child, a lack of knowledge of maternal health care, and inter-province migration. The low rate was found after controlling for socio-demographic, reproductive, and migrant characteristics in this study. The results of a previous study revealed three reasons why women did not participate in PCC: perceived knowledge, perceived lack of risk, and a misunderstanding of the aim of PCC [27]. Younger women (20–24 years) may have thought they were healthy and at an appropriate age for conception, and hence, were not at risk. Thus, they tended not to use PCC. Families with more than one child also had a low rate of PCC, perhaps because of previous experience with pregnancy and higher levels of confidence in handling pregnancy. Consistent with the report by Zhu et al., primigravid women were more likely to attend a preconception clinic [25]. Women who had a better knowledge of maternal health care were more likely to receive PCC. An in-depth knowledge of pregnancy may increase women’s understanding and awareness of the purpose and importance of PCC, and thus, their use of this service. In our study, knowledge of maternal health care was assessed only by two questions measuring appropriate times for prenatal care and symptoms of anemia during pregnancy, which are important components of maternal health care. However, it was only a partial assessment of maternal health care. Future studies focusing on knowledge of maternal health should include additional questions. People in the same province usually had access to the same health care system and used the same maternal health care services, such as the suggested number of prenatal care visits [28]. In addition, those residing in the same province may share the same culture, while individuals across diverse provinces may differ culturally from one another [29]. This study found that local women had a higher rate of receiving PCC (28.0%). These findings may explain why migrants from other provinces had lower rates of PCC.

The positive association between receiving PCC and taking preconception folic acid supplements in our study is consistent with the findings of other studies. Previous studies found that the use of PCC was significantly related to women’s use of and compliance with taking folic acid supplements [30-32]. We also found that PCC was significantly associated with quality prenatal care. This result is consistent with the findings of Williams et al., who found that PCC was positively associated with first trimester entry into prenatal care among women with intended pregnancies (AOR = 2.05) [26]. As described in the measures section of this report, quality prenatal care was defined by several criteria, including entry into prenatal care during the first trimester. The effect of PCC on quality prenatal care was strengthened among migrant women who had been living in Nanhai or Pinghu for less than 5 years. However, the effect disappeared among women who had been living in Nanhai or Pinghu for more than 5 years. This result indicates that more effort should be focused on newly settled migrants to increase quality prenatal care through providing PCC.

The positive association between PCC and maternal health care found in this study suggests that a more comprehensive understanding of the characteristics associated with service use is necessary before designing methods to improve maternal health care. As our study was cross-sectional, prospective or intervention studies are needed to further explore the effect of PCC on maternal health care.

### Strengths and limitations of the study

This novel study focused on factors related to PCC uptake and the effects of PCC on maternal health care in migrants of China. The survey was conducted rigorously using standardized protocols based on the Maternal, Newborn, and Child Health Household Survey Guidelines (WHO, [21]). Data collectors, as well as quality control and other personnel, received training prior to the study. Quality control monitoring was performed throughout the study. The questionnaire was pilot tested before it was used in fieldwork.

Nonetheless, the survey had some limitations. First, the data consisted of retrospective self-reports, which increased the likelihood of recall bias. However, an effort was made to reduce recall bias by analyzing the data on the most recent pregnancy within 1 year of the survey. There is evidence that major life events, such as pregnancy and the birth of a child, might enhance the accuracy of retrospective recall, thus reducing the chance of recall bias [33]. Second, the survey was only based on two cities with the highest proportions of migrants in China. In order to obtain a clearer picture, future research should be expanded into other areas, considering the variations across geographic regions. Third, we acknowledge a need to exercise caution in making a conclusion about causality based on a cross-sectional survey of this kind. Fourth, although this study attempted to control for the potential characteristics associated with PCC, it could not control for all of the variables, such as whether the pregnancy was intended. Fifth, the variable “preconception folic acid supplements” may not fully reflect the preconception use of a folic acid supplement, because we did not collect information about the quantity of folic acid supplements. The measurement of the key indicator PCC might not have been objectively assessed by the question “Before your latest baby was conceived, did you consult with a doctor, nurse, or other health care provider about pregnancy?” Although efforts were made to make this information accurate by questioning the exact time of consultation, this method may have underestimated the real prevalence of PCC. We also tried to get information about PCC through medical records or provider reports. However, the earliest written record of prenatal care we have is the “registration card for pregnancy”, which suggested finishing registration during the first trimester.

## Conclusions

The prevalence of PCC among migrant women was low (20.6%, 95% CI: 18.1–23.1%). Younger age (20–24 years), having more than one child, lack of knowledge of maternal health care, and inter-province migration were predictors of a lack of PCC. Positive associations were found between the receipt of PCC and preconception folic acid supplements and quality prenatal care. Among migrant women who had been living in Nanhai or Pinghu for less than 5 years, the positive association between PCC and quality prenatal care was strengthened. Future studies on maternal health care should focus attention on PCC and explore the effects of PCC on maternal health care through intervention studies. Continued efforts to increase PCC in migrant women should target specific age groups (20–24 years), families with more than one child, and women who have migrated between provinces, as well as provide in-depth knowledge of maternal health care.

## Abbreviations

AOR: Adjusted odds ratio
CI: Confidence interval
PCC: Preconception counseling
WHO: World Health Organization

## References

[1] Chen J (2011). Internal migration and health: re-examining the healthy migrant phenomenon in China. Soc Sci Med.

[2] National Bureau of Statistics of the People’s Republic of China. Main data of the sixth national population census. http://www.stats.gov.cn/ztjc/zdtjgz/zgrkpc/dlcrkpc/ (in Chinese).

[3] Hu X, Cook S, Salazar MA (2008). Internal migration and health in China. Lancet.

[4] Yuan B, Qian X, Thomsen S (2013). Disadvantaged populations in maternal health in China who and why?. Glob Health Action.

[5] Almeida LM, Caldas J, Ayres-de-Campos D, Salcedo-Barrientos D, Dias S (2013). Maternal healthcare in migrants: a systematic review. Matern Child Health J.

[6] Kusuma YS, Kumari R, Kaushal S (2013). Migration and access to maternal healthcare: determinants of adequate antenatal care and institutional delivery among socio-economically disadvantaged migrants in Delhi, India. Trop Med Int Health.

[7] Lauria L, Bonciani M, Spinelli A, Grandolfo ME (2013). Inequalities in maternal care in Italy: the role of socioeconomic and migrant status. Ann Ist Super Sanita.

[8] Singh PK, Rai RK, Singh L (2012). Examining the effect of household wealth and migration status on safe delivery care in urban India, 1992–2006. PLoS One.

[9] Bircher H (2009). Prenatal care disparities and the migrant farm worker community. MCN Am J Matern Child Nurs.

[10] Zhan SK, Sun ZW, Blas E (2002). Economic transition and maternal health care for internal migrants in Shanghai, China. Health Policy Plan.

[11] Liang J, Dai L, Zhu J, Li X, Zeng W, Wang H (2011). Preventable maternal mortality: geographic/rural–urban differences and associated factors from the population-based Maternal Mortality Surveillance System in China. BMC Public Health.

[12] Walfisch A, Koren G (2011). Preconception counseling: rational, practice and challenges. Minerva Ginecol.

[13] Ministry of Health (2007). The job specification of preconception care (Trial).

[14] Qi H, Chang Q, Li L (2011). Guideline for prenatal and antenatal health care. Chinese J Obstet Gynecol.

[15] Ministry of Health (2011). Management practices and norms of maternal health care.

[16] Zhao X, Jiang X, Zhu J, Li G, He X, Ma F (2014). Factors influencing the quality of preconception healthcare in China: applying a preconceptional instrument to assess healthcare needs. BMC Pregnancy Childbirth.

[17] Chandranipapongse W, Koren G (2013). Preconception counseling for preventable risks. Can Fam Physician.

[18] Kermack AJ, Macklon N (2013). Preconception care and fertility. Minerva Ginecol.

[19] Czeizel AE (2012). Experience of the Hungarian Preconception Service between 1984 and 2010. Eur J Obstet Gynecol Reprod Biol.

[20] Wahabi HA, Alzeidan RA, Bawazeer GA, Alansari LA, Esmaeil SA (2010). Preconception care for diabetic women for improving maternal and fetal outcomes: a systematic review and meta-analysis. BMC Pregnancy Childbirth.

[21] WHO (2009). Maternal, newborn and child health household survey guidelines (Draft).

[22] Chen H, Xu H (2013). The current situation, problem, and strategy for primary prevention of birth defects in Fengxian district in Shanghai city. Maternal Child Health Care China.

[23] Ren Z (2011). Utilisation of antenatal care in four counties in Ningxia, China. Midwifery.

[24] Gyampoh S, Otoo GE, Aryeetey RN (2014). Child feeding knowledge and practices among women participating in growth monitoring and promotion in Accra, Ghana. BMC Pregnancy Childbirth.

[25] Zhu H, Graham D, Teh RW, Hornbuckle J (2012). Utilisation of preconception care in women with pregestational diabetes in Western Australia. Aust N Z J Obstet Gynaecol.

[26] Williams L, Zapata LB, D’Angelo DV, Harrison L, Morrow B (2012). Associations between preconception counseling and maternal behaviors before and during pregnancy. Matern Child Health J.

[27] Hosli EJ, Elsinga J, Buitendijk SE, Assendelft WJ, van der Pal-de BK (2008). Women’s motives for not participating in preconception counseling: qualitative study. Community Genet.

[28] Tang M, Wang D, Hu H, Wang G, Li R. Regional differences of maternal health care utilization in China. Asia Pac J Public Health 2013; May 10. doi:10.1177/1010539513487011. 10.1177/1010539513487011 23666840

[29] Lyberg A, Viken B, Haruna M, Severinsson E (2012). Diversity and challenges in the management of maternity care for migrant women. J Nurs Manag.

[30] Pastuszak A, Bhatia D, Okotore B, Koren G (1999). Preconception counseling and women’s compliance with folic acid supplementation. Can Fam Physician.

[31] Elsinga J, de Jong-Potjer LC, van der Pal-de BK, le Cessie S, Assendelft WJ, Buitendijk SE (2008). The effect of preconception counselling on lifestyle and other behaviour before and during pregnancy. Womens Health Issues.

[32] Agricola E, Pandolfi E, Gonfiantini MV, Gesualdo F, Romano M, Carloni E (2014). A cohort study of a tailored web intervention for preconception care. BMC Med Inform Decis Mak.

[33] Belli RF (1998). The structure of autobiographical memory and the event history calendar: potential improvements in the quality of retrospective reports in surveys. Memory.

